# Helicobacter Pylori Promote B7-H1 Expression by Suppressing miR-152 and miR-200b in Gastric Cancer Cells

**DOI:** 10.1371/journal.pone.0168822

**Published:** 2017-01-05

**Authors:** Gengchen Xie, Wei Li, Ruidong Li, Ke Wu, Ende Zhao, Yu Zhang, Peng Zhang, Liang Shi, Di Wang, Yuping Yin, Rui Deng, Kaixiong Tao

**Affiliations:** 1 Department of Gastrointestinal Surgery of Union Hospital, Tongji Medical College, Huazhong University of Science and Technology, Wuhan, Hubei, P.R. China; 2 Department of Radiology of Union Hospital, Tongji Medical College, Huazhong University of Science and Technology, Wuhan, Hubei, P.R. China; 3 Department of Clinical Laboratory of Union Hospital, Tongji Medical College, Huazhong University of Science and Technology, Wuhan, Hubei, P.R. China; New York State Institute for Basic Research, UNITED STATES

## Abstract

The most common cause of gastric cancer is infection with helicobacter pylori (HP), but the associated molecular mechanism is not well understood. In the present study, we found a marked increase in the expression of B7-H1, a member of the B7 co-stimulatory family of molecules that bind to programmed death-1 (PD-1) and play a critical immunoregulatory role in the cell-mediated immune response, in HP-positive gastric cancer tissue. Infection of cultured gastric cancer cells with HP promoted B7-H1 expression and inhibited miR-152 and miR-200b expression. We further demonstrated that these two miRNAs targeted B7-H1 mRNA and suppressed B7-H1 expression in gastric cancer cells. Finally, B7-H1 expression was found to correlate with miR-152 and miR-200b levels in gastric tumor tissues from human patients. Our findings suggest a novel mechanism by which HP infection promotes gastric cancer and also suggest potential targets, i.e., miR-152 and miR-200b, for the prevention and treatment of gastric cancer.

## Introduction

Gastric cancer is the second leading cause of cancer-associated death worldwide. For decades, cancer researchers have wondered why the immune system cannot recognize tumor cells as invaders and kill these cells. Immune escape plays an important role in tumor progression [1], and immune suppression is the primary mechanism underlying tumor immune escape. A recent study suggested that the main cause of immune escape in gastric cancer could be the apoptosis and functional inhibition of immune cells that are induced by cancer cells [2].

B7-H1 (PD-L1; CD274) is a novel B7 family member that exhibits important suppressive functions in the cell-mediated immune response by inhibiting the proliferation of T cells [3]. B7-H1 forms and maintains an immunosuppressive microenvironment by inhibiting the proliferation of activated T cells and inducing the apoptosis of T cells [4]. B7-H1 is over-expressed in tumor cells compared with normal gastric epithelial cells [5–7]. Increased B7-H1 expression has also been detected in human gastric epithelial cells in cases of Helicobacter pylori (HP) infection [8]. HP can upregulate B7-H1 expression by activating the p38 MAPK pathway, thus to establish a persistent infection characteristic of HP [9]. B7-H1 is a ligand of the programmed death-1 (PD-1) receptor, which delivers inhibitory signals to T cells to inhibit immune responses [10]. The ablation of the B7-H1 and PD-1 interaction with blocking antibodies can restore cytotoxic T lymphocyte (CTL)-mediated tumor lysis in vitro, suggesting a novel target for cancer therapy [11].

The most common cause of gastric cancer is infection with the Gram-negative, spiral-shaped bacteria HP, which infects approximately 50% of the world’s population. HP infection leads to chronic inflammation, and the clinical consequences range from gastritis to gastric and duodenal ulcers and gastric malignancy [12,13].

MicroRNAs (miRNAs) are endogenous non-coding RNAs of 21–23 nucleotides that participate in the post-transcriptional regulation of gene expression by pairing with the 3’-untranslated region (3’-UTR) of the messenger RNA (mRNA) of the target gene, which leads to the silencing of the specific gene [14]. The downregulation of the expression of certain tumor-suppressive miRNAs can lead to the over-expression of target proteins, over-proliferation, the inhibition of cancer cell apoptosis and the acceleration of tumor development [15]. It has been reported that B7-H1 expression may be regulated by miR-570 in gastric cancer [16, 17] and by miR-20b, miR-21 and miR-130b in colorectal cancer [18]. Aberrant miRNA expression is involved in the development and progression of gastric cancer. B7-H1 mRNA is constitutively expressed at a very low level, and thus the B7-H1 protein is undetectable under physiological conditions. However, abnormally high expression levels of the B7-H1 protein can be found in malignant tumor tissues, suggesting that B7-H1 expression is deregulated and is involved in cancer. However, the molecular mechanism of the deregulation of B7-H1 expression in gastric cancer remains elusive.

In the present study, we investigated the mechanism whereby HP promotes B7-H1 expression through miR-152 and miR-200b.

## Materials and Methods

### Gastric cancer tissue samples

Gastric cancer tissue samples were collected from gastric cancer tissue removed from patients by surgery without identification of the patients’ personal information at the Union Hospital of Huazhong University of Science and Technology, Hubei Province, China during 2015. The tissue samples were immediately snap frozen in liquid nitrogen, and stored at −80°C. Some tissue samples were fixed in 4% paraformaldehyde, and frozen sections were prepared for immunofluorescence staining. The samples were identified as HP-positive when a rapid urease test was positive. These patients were also confirmed using a 13C breath test. The use of human cancer tissue in this study was reviewed and approved by the Research Ethics Committee of the Tongji Medical College of Huazhong University of Science and Technology. Consent, both written and oral, was obtained before the samples were collected. Authors had not access to information that could identify individual participants during or after data collection.

### Cell culture

Human gastric carcinoma (AGS) cells were obtained from American Type Culture Collection (ATCC; Manassas, VA, USA) and cultured in RPMI 1640 medium supplemented with 10% heat-inactivated fetal bovine serum (FBS) in a 5% CO_2_ atmosphere at 37°C. For the assays described herein, a cell suspension was placed in each well of a flat-bottom 6-well CellBind plate (Corning Inc., Corning, NY, USA), and the plate was incubated at 37°C in a 5% CO_2_ atmosphere for 2–3 days.

### Bacterial culture

NCTC11637, a CagA-positive strain of HP was purchased from ATCC. HP cells were routinely grown on tryptic soy agar (BD 236950) plates supplemented with 5% sheep blood in mixed air containing 10% CO_2_, 5% O_2_ and 8% N_2_ at 37°C.

### Infection of AGS cells with HP

Before infecting AGS cells with HP, the AGS cells were washed and resuspended in fresh antibiotic-free medium. The bacteria were cultured in tryptic soy broth (BD 211825) with 5% sheep blood in mixed air containing 10% CO_2_, 5% O_2_ and 8% N_2_ at 37°C with agitation (200 rpm) for 48 h, harvested and resuspended in RPMI 1640 medium. The AGS cells were maintained in RPMI 1640 with 10% FBS. Cells grown to 90% confluency were infected with HP at an HP/cell ratio of 10/1. HP was added to 6-well cell culture plates. The cells were co-cultured with HP at 37°C in a humidified atmosphere before harvesting.

### Flow cytometry

Flow cytometry was used to assess B7-H1 staining on the surface of cultured AGS cells. Samples were collected after a 24-h incubation with or without bacteria. Prior to performing flow cytometry, cells were harvested, counted and washed. The cells were washed again and incubated with the anti-B7-H1 antibody or with isotype controls for 30 min in the dark at room temperature. After immunostaining, the cells were washed twice with PBS and fixed with paraformaldehyde (1% in PBS). The cells were analyzed via flow cytometry with an LSRII instrument, for which at least 104 live events were analyzed with the cultured human AGS cells to obtain a 90% confidence interval. The data were analyzed using BD FACSDiva (BD Biosciences, San Jose, CA) and FlowJo (Tree Star, Inc., Ashland, OR) software.

### Luciferase assays

The 3′UTR of B7-H1 mRNA containing the miR-152 and miR-200b target sequences were cloned into the XhoI and NotI sites of psiCHECK-2 (Promega) using the primers B7-H1 3’UTR F1 and B7-H1 3’UTR R1 to generate a B7-H1 reporter. The Luciferase activity in the AGS cells was evaluated using the Dual-Glo Luciferase Assay System (Promega) according to the manufacturer's instructions. The luminescence signal was measured with a VICTOR3 Multilabel Counter (Perkin-Elmer, Waltham, MA). The relative percentages of luminescence intensity were calculated by comparison with a MOCK-treated control.

### Immunofluorescence technique

The sections were permeabilized with 0.1% saponin for 30 min and blocked with 5% normal goat serum (NGS) for 30 min. The sections were also permeabilized for 30 min with 0.1% Triton X. The sections were then incubated overnight at 4°C with a primary antibody followed by incubation with a fluorescently labeled secondary antibody and mounting with Hoechst (Invitrogen Life Technologies, Grand Island, NY, USA). The primary antibodies used in the experiments were anti-human B7-H1 (Invitrogen) and anti-human Epcam (Invitrogen). The secondary rabbit anti-mouse FITC and rabbit anti-mouse PE antibodies were purchased from Invitrogen Life Technologies.

Fluorescently immunolabeled sections were imaged with a confocal laser scanning microscope (Leica TCS SP8; Leica Microsystems, Mannheim, Germany). To evaluate the labeling, confocal scanning was performed at 40× magnification. The staining density was analyzed using Image-Pro Plus 4.5 (Media Cybernetics, Silver Spring, MD, USA).

### RNA extraction and quantitative real-time PCR

Total RNA from tissue samples and cultured cells was extracted using the TRIzol reagent (Invitrogen). Quantitative real-time PCR (qRT-PCR) assays were carried out to detect mRNA expression using the PrimeScript RT Reagent Kit (TaKaRa) and SYBR Premix Ex Taq (TaKaRa) according to the manufacturer’s instructions. The expression levels of mature miR-152 and miR-200b were measured with TaqMan miRNA assays (Applied Biosystems) according to the protocol provided, and U6 small nuclear RNA was used as an internal control.

### Target gene prediction

Three computational algorithms, PicTar (http://pictar.mdc-berlin.de/) [19],TargetScan (http://www.targetscan.org/) [20], and MiRanda (http://www.microrna.org) [21], were employed to predict the miRNA targets, and the genes predicted by at least 2 independent tools were considered.

### Statistical analysis

Each experiment was repeated at least in triplicate. The data are presented as the mean ± standard deviation. The data were analyzed using Student’s t-test and one-way analysis of variance. All the statistical analyses were performed using SPSS version 17.0 software (SPSS, Inc., Chicago, IL, USA). A p value < 0.05 was considered to represent statistical significance.

## Results

### B7-H1 expression is increased in HP-positive gastric cancer tissue

**Fig 1:**
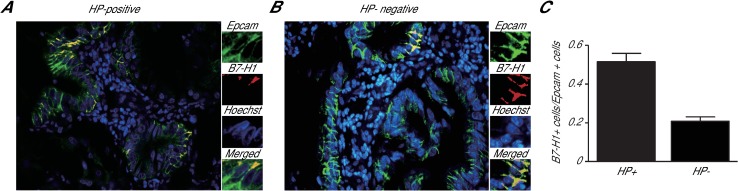
B7-H1 levels in the HP-positive and HP-negative gastric cancer tissue samples. (A,B) HP+ and HP- gastric cancer tissue samples were double-immunostained with antibodies against Epcam (green) and B7-H1 (red). Hoechst staining (blue) was used to label the nuclei. (C) The ratio of B7-H1-positive cells to Epcam-positive cells in the HP+ (n = 76) and HP- (n = 20) gastric cancer tissue samples.

To investigate whether HP infection is involved in the B7-H1-associated carcinogenesis of gastric cancer, we measured B7-H1 levels in HP-positive and HP-negative gastric cancer tissue samples using double immunofluorescence. We observed a much greater number of B7-H1-positive cancer cells in the HP-positive samples than in the HP-negative samples, as marked by the double immunostaining of both B7-H1 and Epcam, a marker for gastric cancer cells (Fig 1A and 1B). A quantitative analysis revealed that 51.5±4.3% and 20.8±2.2% of the cancer cells (Epcam+ cells) were B7-H1 positive in the HP-positive and HP-negative samples, respectively (Fig 1C). A specimen with ≥10% B7-H1-positive tumor cells was classified as B7-H1-positive [22], and 49 of 76 HP-positive specimens (64.5%) were B7-H1 positive, whereas only 7 of 20 HP-negative specimens (35.0%) were found to be B7-H1 positive (Table 1). The less differentiated gastric cancer samples and the samples from patients at a higher TNM stage also exhibited a higher B7-H1-positive rate. These results suggest that HP infection may be associated with B7-H1 expression in gastric cancer and that B7-H1 expression is related to the invasion and malignancy of gastric cancer cells.

**Table 1:** B7-H1 positive rate in clinical samples.

Characteristics	n	B7-H1-positive (%)	*P* value
Gender			0.263
Male	56	30 (53.6)	
Female	40	26 (65.0)	
Age			0.313
≥ 60 years	26	13 (50.0)	
< 60 years	70	43 (61.4)	
HP+			0.017
Yes	76	49 (64.5)	
No	20	7 (35.0)	
Differentiation			0.730
High	27	15 (55.6)	
Low	69	41 (59.4)	
TNM stage			0.012
I	8	3 (37.5)	
II	19	9 (47.3)	
III	39	22 (56.4)	
IV	30	22 (73.3)	

Samples were defined as B7-H1-positive if the ratio of B7-H1-positive cells to Epcam-positive cells in the specimen was ≥10%.

### HP promotes B7-H1 expression in gastric cancer cells

**Fig 2:**
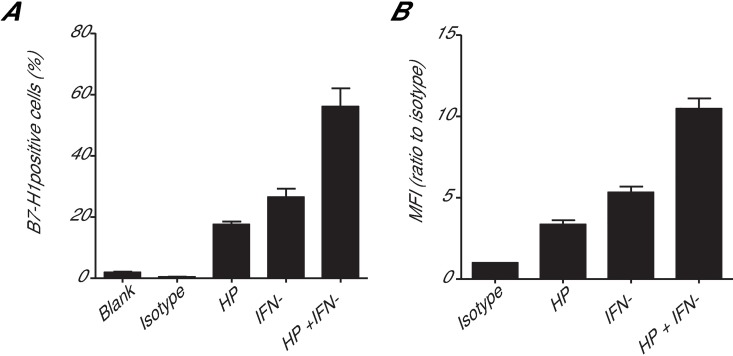
Cytometric analysis of AGS cells after HP infection and/or IFN-γ treatment. B7-H1 expression in AGS cells was determined by flow cytometry after co-culturing the cells with HP (HP/cell ratio: 100/1) and/or treating the cells with IFN-γ (10 ng/ml) for 24 h. (A) Quantification of the cytometric data shows the percentages of B7-H1-positive AGS cells. (B) The ratio of the MFI (mean fluorescence intensity) of each group to the MFI of the isotype group.

To investigate the effects of HP on B7-H1 expression in gastric cancer cells, we infected cultured human gastric carcinoma AGS cells with HP and then measured the surface expression of B7-H1 using flow cytometry. We found a marked increase in B7-H1 expression on the surface of the AGS cells 24 h after infection with an HP strain (Fig 2). Because interferon-γ (IFN-γ) can be produced within the HP-infected gastric mucosa [23], which might also stimulate B7-H1 expression [24], we investigated whether IFN-γ could modulate HP-mediated B7-H1 expression in AGS cells. We found that the IFN-γ treatment also markedly stimulated B7-H1 expression in AGS cells (Fig 2). Furthermore, IFN-γ and HP had an additive effect on stimulating surface B7-H1 expression in the AGS cells. These observations confirm that HP infection can stimulate B7-H1 expression in human gastric cancer cells.

### HP inhibits miRNA expression in gastric cancer cells

**Fig 3:**
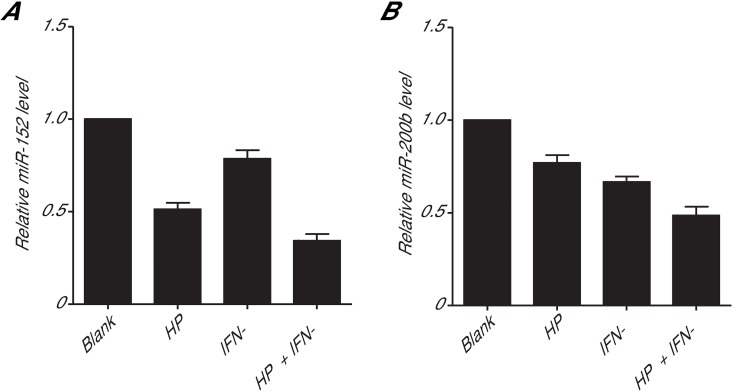
Levels of miR-152 and miR-200b in gastric cancer cells after HP infection or IFN-γ treatment. AGS cells were co-cultured with HP (HP/cell ratio: 100/1) and/or treated with IFN-γ (100 ng/ml) for 24 h, followed by qPCR to measure the levels of miR-152 (A) and miR-200b (B) in cell lysates.

It has been recently demonstrated that B7-H1 expression in cancer cells is regulated by miRNAs [16, 17]. To determine whether the HP-induced increase in B7-H1 expression in human gastric cancer cells occurs via miRNAs targeting B7-H1, we studied two candidate B7-H1 miRNAs (miR-152 and miR-200b) that were predicted byTargetScan and PicTar in AGS cells after HP infection. We found that HP infection reduced both miR-152(Fig 3A) and miR-200b (Fig 3B). In addition, the treatment of AGS cells with IFN-γ, which is produced by AGS cells after HP infection, also reduced the levels of these two miRNAs. An additive effect was observed with both HP infection and IFN-γ treatment. These results suggest that HP infection may stimulate B7-H1 expression through the inhibition of miR-152 and miR-200b in gastric cancer cells.

### MiR-152 and miR-200b target B7-H1 and suppress B7-H1 expression in gastric cancer cells

**Fig 4:**
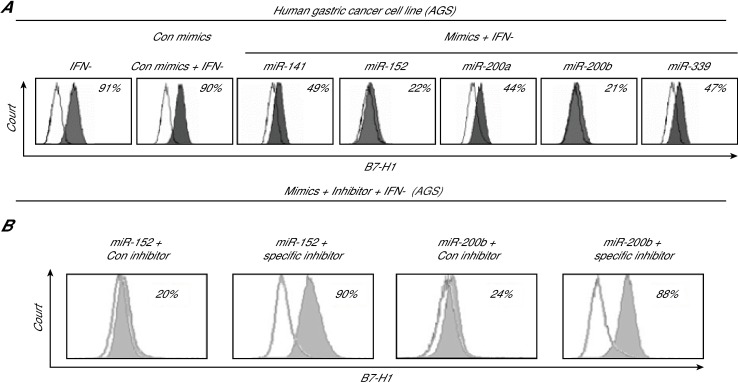
B7-H1 expression in AGS cells after treatment with mimics or inhibitors of miR-152 and miR-200b. (A) AGS cells were transfected with various miRNA mimics for 48 h, and the cells with surface B7-H1 expression were sorted by flow cytometry. (B) AGS cells were treated with various miRNA inhibitors for 48 h, and the cells containing surface B7-H1 expression were sorted by flow cytometry.

To investigate the role of miR-152 and miR-200b in the elevation of B7-H1 expression in AGS cells, we treated the IFN-γ-treated AGS cells with several miRNAs, including miR-152 and miR-200b, and then determined the level of B7-H1 on the surface of the treated cells using flow cytometry. As expected, the IFN-γ treatment led to B7-H1 expression in ~90% of the cells (Fig 4A). In contrast to transfection with the control nucleotides, transfection of the cells with miR-152 and miR-200b markedly inhibited B7-H1 expression, leading to only 21–22% of the cells expressing B7-H1 with IFN-γ treatment. Other miRNAs had a partial effect on silencing B7-H1 expression. In contrast, the treatment of the cells with inhibitors of miR-152 and miR-200b increased the expression of B7-H1 (Fig 4B). These results indicate that miR-152 and miR-200b indeed suppress B7-H1 expression in AGS cells, and support our conclusion that HP infection promotes B7-H1 expression through the downregulation of miR-152 and miR-200b in gastric cancer cells.

**Fig 5:**
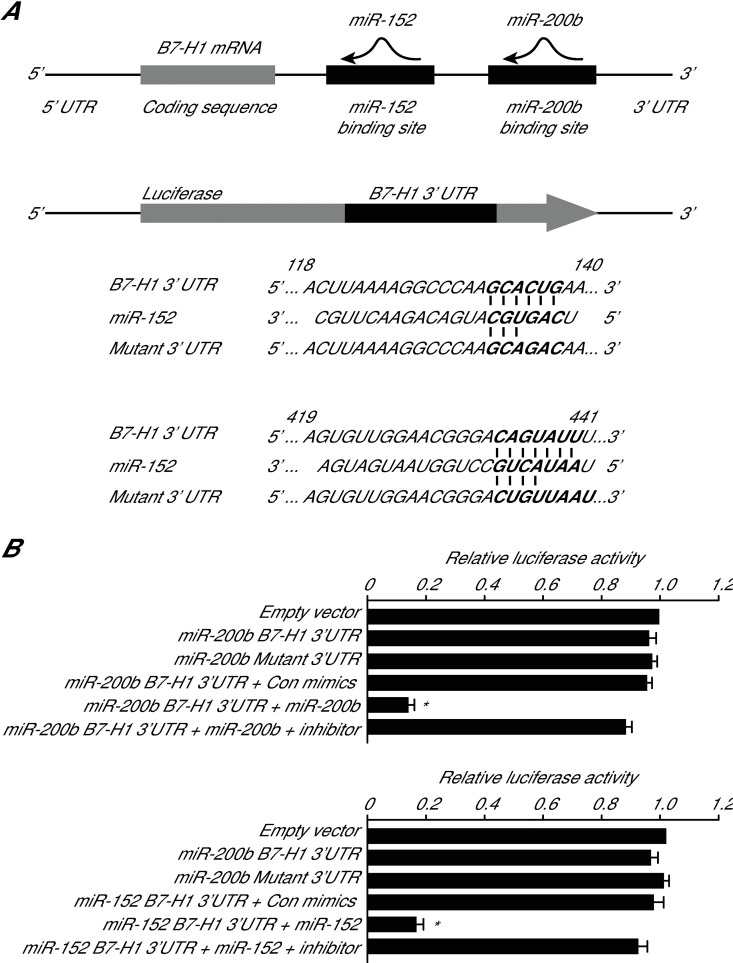
Binding of miR-152 and miR-200b to B7-H1 mRNA. (A) Schematic of B7-H1 mRNA showing potential miR-152 and miR-200b binding sites in the 3’-UTR and schematic of the luciferase reporter. The complementary miR-152 and miR-200b binding sites in the B7-H1 3’ UTR were inserted downstream of a luciferase reporter. (B) Targeting of the B7-H1 mRNA 3’-UTR by miR-152 or miR-200b results in translational suppression. * p<0.05.

To confirm the regulation of B7-H1 expression by miR-152 and miR-200b, we investigated using a luciferase reporter system whether these miRNAs can bind to the 3' UTR of B7-H1 mRNA and inhibit its translation (Fig 5A). We found that the luciferase activity was not affected after transfection of the AGS cells with empty vector, B7-H1 3’UTR, mutant B7-H1 3’UTR, or B7-H13’UTR plus control nucleotides (Fig 5B). In the miR-152 and miR-200b plus B7-H13’UTR groups, the luciferase activity was significantly inhibited. However, this effect was markedly eliminated when inhibitors of miR-152 and miR-200b were transfected together. These results indicate that both miR-152 and miR-200b can bind to the 3’ UTR of B7-H1 mRNA (Fig 5B).

### B7-H1 expression correlates with the levels of miRNAs in gastric tumor tissues

**Fig 6:**
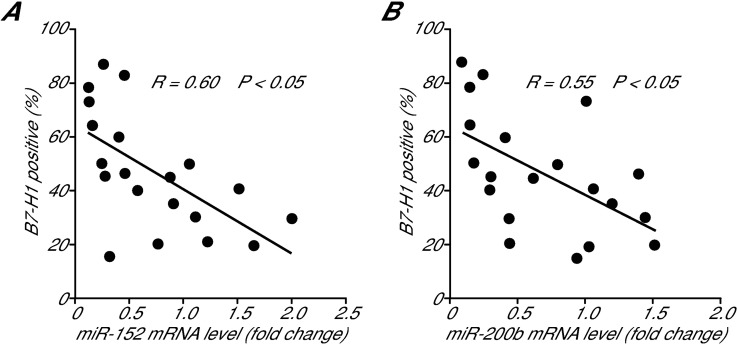
Correlation between the B7-H1-positive rate and miR-152 and miR-200b levels in human gastric cancer tissue samples. The percentages of B7-H1-positive cancer cells, as determined by double immunofluoresence, in 20 human gastric cancer samples were plotted against the levels of miR-152 (A) and miR-200b (B), as determined by qPCR. The linear regression was analyzed using SPSS.

We determined the levels of miR-152 (Fig 6A) and miR-200b (Fig 6B) in 20 human gastric cancer samples using a qPCR assay and then analyzed their correlation to B7-H1 expression levels. We found a negative correlation between the B7-H1 expression levels and these two miRNA levels (Fig 6). These results further support our conclusion that HP infection promotes B7-H1 expression via the downregulation of miR-152 and miR-200b in human gastric cancer cells.

## Discussion

According to previous studies, B7-H1 can affect the immune escape of cancer cells [25]. B7-H1 causes a negative regulation of the immune response by inhibiting T cell activation and activated T cell function and survival [25–28]. Moreover, emerging evidence suggests that the functions of B7-H1 in T cells may be important in regulating the host anti-microbial immune response. Furthermore, the application of anti-B7-H1 and anti-PD-1 antibodies in vitro significantly promoted T-cell activity [25, 29, 30]. In the present study, we found that B7-H1 expression was significantly higher in HP-positive gastric cancer tissues compared with HP-negative gastric cancer tissues and that the infection of AGS cells with HP induced B7-H1 expression. Therefore, the expression of B7-H1 in HP-infected gastric cancer may play a key role in T cell immunity in gastric carcinogenesis. Furthermore, B7-H1 is an indicator of poor prognosis in patients with renal cell carcinoma [31], esophageal cancer [22], gastric carcinoma [7], breast cancer [32], ovarian cancer [33], bladder urothelial carcinoma [34], or pancreatic cancer [35]. It has been confirmed that the extent of B7-H1 expression in gastric cancer is significantly related to the clinicopathological features, including tumor size, depth of invasion, lymph node metastasis, and prognosis of patients [7, 17]. The results of our study demonstrated that B7-H1 expression in gastric cancer was associated with tumor malignancy; that is, the higher the expression of B7-H1, the lower the differentiation of the gastric cancer and the higher the TNM stage. The results indicate that B7-H1 may be a positive element for tumor invasion and metastasis in gastric cancer patients with HP infections.

In most of the cancer cells and epithelial cells, B7-H1 expression cannot be activated without the stimulation of IFN-γ. The regulation of IFN-γ on B7-H1 expression is at the transcriptional level[36]. In addition, the B7-H1 is also regulated post-transcriptionally. If cells are treated with IFN-γ at a high dose (≥50 ng/mL), the regulation of miRNAs on B7-H1 expression disappears, probably because the amount of miRNA is insufficient for down-regulating the large amount of B7-H1 mRNA induced by high dose of IFN-γ.

Many studies have indicated that miR-152 and miR-200b potentially function as tumor suppressors and are downregulated in various tumor types. The downregulation of miR-152 induces abnormal DNA methylation in HBV-related hepatocellular carcinoma (HCC) by inhibiting DNA methyltransferase 1 (DNMT1) expression [37]. MiR-152 acts as a tumor suppressor that reduces the migratory and invasive capabilities of prostate cancer cells by targeting TGF-á [38]. Moreover, miR-152 is significantly reduced in ovarian cancer cells, and miR-152 regulates ovarian cancer cisplatin resistance by targeting DNMT1 [39]. MiR-200b, a member of the miRNA-200 family, also functions as a tumor suppressor in a wide range of human malignances, including breast, colorectal and pancreatic cancer [40–42]. Our data indicated that HP inhibited miR-152 and miR-200b expression in gastric cancer cells, and miR-152 and miR-200b target B7-H1 and suppress B7-H1 expression in gastric cancer cells. Thus, miR-152 and miR-200b function as important tumor suppressors in HP-related gastric cancer.

In our study, we confirmed that B7-H1 expression can be upregulated by HP infection in gastric cancer cells. Thus, B7-H1 may act as important regulator in controlling and balancing inflammatory reactions in HP-related gastric cancer development. We found that miR-152 and miR-200b expression levels were significantly decreased in HP-related gastric cancer and that miR-152 and miR-200b suppressed B7-H1 expression in gastric cancer cells. These results suggest that miR-152 and miR-200b might play an essential role in gastric carcinogenesis and that HP infection may downregulate miR-152 and miR-200b expression resulting in a significant upregulation of B7-H1 expression in gastric cancer. A previous study revealed that the promoters of a variety of miRNA genes are hypermethylated in gastric cancer DNA, and these miRNAs act as suppressors of cancer development [43], indicating that the methylation of the promoters of miRNAs leads to the silencing of certain miRNAs in the pathogenesis of gastric cancer. It can be postulated that the hypermethylation of miRNA promoters caused by HP stimulating DNMT1 expression through the AKT-NFκB pathway may be the mechanism whereby HP downregulates miR-152 and miR-200b, which requires further investigation.

Taken together, the findings of our study suggest that B7-H1 expression in gastric cancer cells can be upregulated by HP infection via the suppression of miR-152 and miR-200b. It is of interest whether these findings can be extended beyond the HP infection of gastric cancer cells. Further studies should also determine the mechanisms by which HP infection decreases miR-152 and miR-200b expression and the role of miRNAs in host anti-HP immunity.
